# *PolyMorphPredict*: A Universal Web-Tool for Rapid Polymorphic Microsatellite Marker Discovery From Whole Genome and Transcriptome Data

**DOI:** 10.3389/fpls.2018.01966

**Published:** 2019-01-11

**Authors:** Ritwika Das, Vasu Arora, Sarika Jaiswal, MA Iquebal, UB Angadi, Samar Fatma, Rakesh Singh, Sandip Shil, Anil Rai, Dinesh Kumar

**Affiliations:** ^1^Centre for Agricultural Bioinformatics, ICAR-Indian Agricultural Statistics Research Institute, New Delhi, India; ^2^ICAR-National Bureau of Plant Genetic Resources, New Delhi, India; ^3^Research Center, ICAR-Central Plantation Crops Research Institute, Jalpaiguri, India

**Keywords:** DUS, genic region microsatellite, markers, polymorphism, web server

## Abstract

Microsatellites are ubiquitously distributed, polymorphic repeat sequence valuable for association, selection, population structure and identification. They can be mined by genomic library, probe hybridization and sequencing of selected clones. Such approach has many limitations like biased hybridization and selection of larger repeats. *In silico* mining of polymorphic markers using data of various genotypes can be rapid and economical. Available tools lack in some or other aspects like: targeted user defined primer generation, polymorphism discovery using multiple sequence, size and number limits of input sequence, no option for primer generation and e-PCR evaluation, transferability, lack of complete automation and user-friendliness. They also lack the provision to evaluate published primers in e-PCR mode to generate additional allelic data using re-sequenced data of various genotypes for judicious utilization of previously generated data. We developed the tool (*PolyMorphPredict*) using Perl, R, Java and launched at Apache which is available at http://webtom.cabgrid.res.in/polypred/. It mines microsatellite loci and computes primers from genome/transcriptome data of any species. It can perform e-PCR using published primers for polymorphism discovery and across species transferability of microsatellite loci. Present tool has been evaluated using five species of different genome size having 21 genotypes. Though server is equipped with genomic data of three species for test run with gel simulation, but can be used for any species. Further, polymorphism predictability has been validated using *in silico* and *in vitro* PCR of four rice genotypes. This tool can accelerate the *in silico* microsatellite polymorphism discovery in re-sequencing projects of any species of plant and animal for their diversity estimation along with variety/breed identification, population structure, MAS, QTL and gene discovery, traceability, parentage testing, fungal diagnostics and genome finishing.

## Introduction

A good molecular marker has attributes like simplicity, abundance/wide genome coverage, ubiquity, inheritance/co-dominance, and multi-allelism or polymorphism among genomes with universality of genotyping in terms of reproducibility and amenability for multiplexing in detection (Powell et al., 1996). Microsatellite are putative DNA markers of simple sequence repeats (SSRs) sequences having tandem repeats (1–6 base pair). In a given population, allelic variation is generated due to inherent thermodynamic properties of repeat sequence leading to out of register replication by slippage event during replication (Victoria et al., 2011). When a particular allele has frequency of >1%, in population it is called polymorphic allele (Jarne and Lagoda, 1996). Since SSR locus has higher mutation rate thus it results into change in allelic array length (by addition or deletion of repeat) and making it a preferred hypervariable codominant markers. It can be used in linkage mapping, QTL and gene discovery, population genetics, pedigree/parentage analysis, phylogenetics and evolutionary studies, gene regulation and genetic disorder (Cavagnaro et al., 2010).

*In vitro* microsatellite marker discovery is expensive, cumbersome or labor intensive and time consuming as it involves creation of a small insert genomic library, screening of positive clones by hybridization, sequencing and designing primers for single locus-specific PCR analysis. Besides this, *in vitro* polymorphism discovery of such markers require throughput genotyping which is again involved with time and cost (Powell et al., 1996; Zhao et al., 2017). With advent of next-generation sequencing (NGS) technology, time and cost in identification of such microsatellite repeat has reduced very drastically thus *in silico* method has replaced the *in vitro* method (Zhao et al., 2017). Microsatellite repeats can be used in PCR based genotyping/fingerprinting for wider applications (Zietkiewicz et al., 1994). Such application also includes origin and identification of cultivar (Testolin et al., 2000).

Previous tool, poly (Bizzaro and Marx, 2003) written in object oriented scripting language Python which gives output having microsatellite locations, tract frequencies and length by command line, thus not user friendly. Pipeline diversity analysis (PDA) (Casillas and Barbadilla, 2004) can search polymorphism in database with genetic diversity estimation without polymorphism and primers for genotyping. SSR analysis tool (SAT) is a web application for SSR search and primer designing but has limitation of manual upload of sequences and having size limitation also Dereeper et al. (2007). There are species specific polymorphic microsatellite database for example, rice having only two varieties (Indica and Japonica) without any option to analyze other rice varieties or e-PCR genotyping (Zhang et al., 2007). QDD (Meglécz et al., 2010) is an open access program for microsatellite discovery and primer designing but takes limited fasta files of <50 K sequence. Though GMATo (Genome-wide Microsatellite Analyzing Tool) tool is powerful and can mine microsatellite of any genome size along with information on statistical distribution of microsatellite in the genome (Wang et al., 2013) but can neither design primers nor detect polymorphism. Though pSTR Finder (Lee et al., 2015) can identify putative polymorphic STR loci using whole genome sequence and compare two genomes in fasta format but result is without allele size and position needed for genotyping/multiplexing. CandiSSR (Xia et al., 2016) is a pipeline to identify candidate polymorphic SSRs based on the multiple assembled sequences of transcriptome/set of genome of a given species or genus but has limitation of standalone mode, complex linux/unix based system besides dependency of other requirements like MISA, Primer3, ClustalW and issues of runtime. PolySSR pipeline (Tang et al., 2008) and GMATo (Wang et al., 2013) were having limitation of standalone mode without having provision of external primer based polymorphism discovery to use and compare with earlier allelic data. DnaSP (Rozas and Rozas, 1995) which is based on coalescent method of polymorphic discovery has great limitation like standalone, input sequence limited to <5 in number and <5 MB size. Genome-wide Microsatellite Analyzing Tool Package (GMATA) integrates microsatellite mining, statistical analysis, plotting, primer designing, polymorphism screening and marker transferability by simulated marker mapping/e-mapping (Wang and Wang, 2016). Being in non-server mode, such standalone tool cannot be handled by biological researchers unless one has specialized computational skill. There is no computational tool where genic region microsatellite discovery can also be done along with primer designing and efficacy of correct designing can be checked immediately using very same tool by conducting e-PCR over whole genome data to avoid intron containing PCR amplicons.

Due to various limitations in existing tools, like some can discover microsatellite but cannot compute primers or discover polymorphism or has size limitation of input sequence. Even some tools have integration of various existing tools without automation. Though few powerful tools are available but they are not available in user friendly server mode. None of the earlier tools can cater the need of chromosome specific targeted region (For example, QTL region) microsatellite polymorphism discovery along with locational details and product size required for various genotyping strategy/multiplexing. There is no such tool having option to use published primers in e-PCR mode to generate additional data and at the same time utilizing the previously generated data very prudently. Our developed tool overcomes on all these limitations, thus our tool is much more pragmatic and prudent in approach.

Present work aims at development of web based tool which can compare various whole genome sequence of any species and their genotypes to discover microsatellite loci and identify polymorphic locus along with designing primers for rapid genotyping. We also aim to develop the tool having provision for evaluation of published primers on various genotypes with in species and transferability between heterologous species in e-PCR mode to generate allelic data for germplasm management and improvement, ecological diversity, population genetics and evolutionary analysis.

## Materials and Methods

*PolyMorphPredict* is designed for mining of microsatellite loci along with primer generation and detection of polymorphism using re-sequence whole genome data or polymorphism discovery by published primers of a given genome/set of genomes/genotypes of any species. All the modules work together irrespective of the languages used like Practical Extraction and Reporting Language, *i.e.*, Perl (64 bit, version 5), R (version 3.0) or Java (version 7) and can be invoked individually independently.

### *PolyMorphPredict* Strategy for Microsatellite Mining and Primer Generation

This module of primer generation integrates *MISA* (Thiel et al., 2003) and *Primer3core* (Untergasser et al., 2012) where MISA mines the microsatellite markers from target sequences (chromosome wise data/sequence in fasta format) provided by the user. The tool can extract flanking sequences of 500 bp upstream and 500 bp downstream of the targeted microsatellite loci from the target reference genome file. In house PERL script was used to aid in parsing the output of MISA for the generation of primers through *Primer3core* integrated herewith. User can change the parameters of MISA, like repeat numbers and maximum distance between two microsatellites. These options can enable to search for potentially higher polymorphic loci to reduce wet-lab cost of polymorphism discovery. Since this tool is designed for rapid microsatellite polymorphism discovery in genome re-sequencing projects where template genome is fully sequenced, thus default parameters of Primer3 has been used to have uniform Tm of all microsatellite loci for bulk genotyping.

### Approach for Polymorphism Detection

This tool is based on the algorithm of allelic length polymorphism detection by computing the amplicon size difference in terms of basepairs. If there is no difference in e-PCR amplicon (product size), then there is no polymorphism between genotypes analyzed. This module of polymorphism detection has two sub-modules for primer selection, namely, self-designed primers (which generates new primers) and External primers (to use already published primers). The Self-Designed Primers module seeks input files of reference genome/chromosome or specific species file and the genotypic files having chromosome data in fasta format. For the ease of users, the web server has two reference model genomes inbuilt, namely, rice and sugarbeet along with small sample dataset of respective species for polymorphism discovery. However, user can upload chromosome-wise genome data of any species along with its re-sequenced data for polymorphism discovery. User can give the number of genotypes of the uploaded species and search for the polymorphic markers followed by uploading of the genotype/transcript files. The list of polymorphic markers will be displayed.

For module “External Primers,” users are given three options to upload input files (fasta format), namely, reference genome file, chromosome file or specific species file. To discover polymorphic markers using the already existing primers, scripts were written in PERL to find the loci having primers in flanking region of the genotype using chromosomal template of a given genome of any species. The e-PCR products having size difference is computed and selected to be reported/displayed as polymorphic loci. Both genomic and transcriptomic microsatellites can be analyzed by this tool. While designing primer especially using transcriptomic data, expected failure due to intron-exon junction can be avoided by prior evaluation using this tool with annotated reference genome.

**FIGURE 1:**
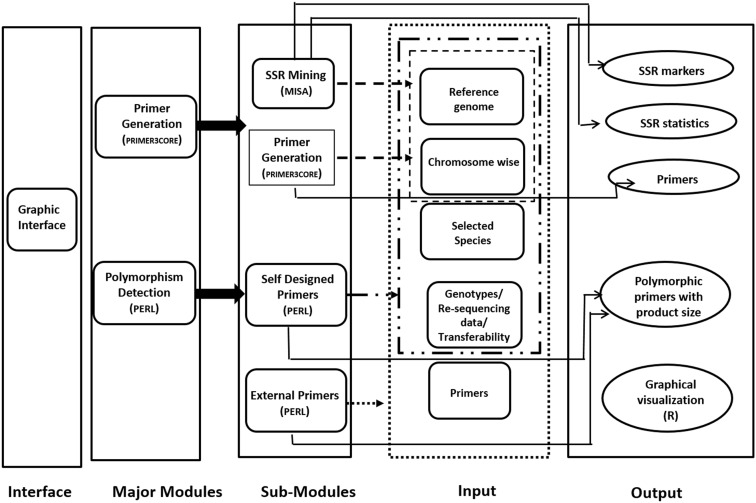
Schematic representation of *PolyMorphPredict* for microsatellite mining, primer designing, and polymorphism discovery.

Provision for simulated gel, developed using R code has been made for ease in development of low cost gel based genotyping strategy. The polymorphic microsatellite markers found in reference and genotypic sequence files can be graphically visualized in the developed web server. Codes are developed using R software which can produce the output as product sizes for the markers for the same primer pair. All the result files can be either downloaded directly or can be obtained by mail also. JavaScript and HTML are used to develop graphical user interface (GUI). This internally calls PERL and R. Only input file(s) of genome/chromosome(s) in plain/fasta format is required for microsatellite mining and generation of primers from these markers. This can be performed by a mouse click in the graphical interface. The workflow of the same is depicted in Figure 1.

### Efficacy of *PolyMorphPredict* for Various Genome Size

In order to evaluate the *in silico* efficacy of this tool across animal and plant kingdom, model species of various genome sizes were selected representing three different levels of genome complexities, namely small (<500 MB), moderate (500 MB–1 GB) and larger genome (>2 GB) which also represented animal and plants. For smaller genome rice and grape (*Vitis*), moderate genome sugarbeet (*Beta vulgaris*) and for larger genome, cattle were used. Genomic data of 21 various genotypes/cultivars representing five species, i.e., rice (6), sugarbeet (5), *Vitis* (5), *Prunus* (2), and cattle (3) were used for microsatellite polymorphism discovery. The details of public domain accessibility of these genomic data is furnished in Supplementary Table S1.

In evaluation of smaller genome rice, having genome size 352 MB, 6 genotypes were used. In this, two cultivars, namely, RP Bio-226 and Shuhui498 were used for *in silico* mining of microsatellite and polymorphism discovery. Remaining four varieties of rice genome (Cauvery, Dubraj, CO36, and CO39) were evaluated for e-PCR based genotyping with known highly variable microsatellite primers followed by *in vitro* PCR to validate the polymorphism discovery (Singh et al., 2013). In case of another smaller genome, grape (∼475 MB), 5 cultivars, namely, PN40024, Chkhaveri, Saperavi, Meskhetian green and Rkatsiteli were used for *in silico* mining of microsatellite and polymorphism discovery. For the moderate genome, sugarbeet (∼750 MB), 5 genotypes, namely, KWS2320, KWS230 DH1440, STR06A6001, SynMono, and SynTilling were used. For evaluation of larger genome represented by cattle (2670.14 MB), three phenotypically distinct breeds from different geographically isolated population, viz., Hereford, Nellore, and Gir were used. While analyzing this larger genome of cattle, the largest chromosome 1 (158.3 MB) was used, where Hereford genome was used as a template for microsatellite polymorphism discovery.

### *In silico* Evaluation for Microsatellite Transferability Across Species

In order to evaluate this module of PolyMorphPredict, public domain data was used. Available sweet cherry (*Prunus avium*) genome was used as focal species and peach (*Prunus persica*) was used as host species. A total of 200 microsatellite markers having simple dinucleotide repeats from chromosome 1 of cherry genome assembly were downloaded^1^. e-PCR was carried out using whole genome sequence of *Prunus* as template. True e-PCR amplicons of microsatellite loci were obtained by filtering out amplicon size of >500 bp and asymmetric match with either forward or reverse primer sequence.

### Validation of *PolyMorphPredict* for Detection of Polymorphism: A Case Study in Rice

In order to evaluate the efficacy of *PolyMorphPredict* for detection of polymorphism, two-step process having *in silico* PCR (e-PCR) followed by *in vitro* PCR using four Indian rice varieties were performed.

#### Polymorphism Discovery by e-PCR

Four genotypes of Indian rice were selected having statutory varietal status also representing within and between diverse ecological habitat, along with their availability in public domain of rice 3K genome project of IRRI, Philippines (Li et al., 2014) for e-PCR and availability of germplasm for *in vitro* PCR. Diverse ecological germplasm panel for validation were selected by taking one representative from ecological regions of central India, Dubraj (Madhya Pradesh, 22.9734° N, 78.6569° E, agro and sub-ecological zone), and another from southern ecological regions India Cauvery (Tamil Nadu, 11.1271° N, 78.6569° E, agro and sub-ecological zone) to evaluate microsatellite polymorphism between ecological regions. For evaluation of polymorphism within same geographically overlapping regions (Tamil Nadu), two additional germplasm namely CO36 and CO39 (Amaravathi) were taken. Whole genome sequence data of these rice varieties were downloaded for assembly and used. *PolyMorphPredict* option “evaluation of External Primer,” was used to perform e-PCR over these genomes to obtain polymorphic loci. Results of polymorphic discovery by *in silico* PCR (e-PCR) and *in vitro* PCR were compared in terms of allele length polymorphism.

#### Polymorphism Discovery by *in vitro* PCR

In order to extract DNA using QIAGEN DNeasy plant mini kit (Hilden, Germany), seeds of each rice variety (10–12 seeds) were dehusked. Fine powder was obtained by grinding kernels using tissue lyser (TissueLyser II Retsch, Germany) with a tissue lyser adapter set (QIAGEN). QIAGEN DNeasy plant mini kit protocol was followed for DNA isolation. DNA samples were checked on 0.8% gel and were quantified using NanoDrop. Dilutions of each DNA samples were prepared to 10 ng/μl and used for PCR reaction.

For genotyping of rice varieties, four microsatellite markers from four different chromosomes were selected from e-PCR result over four different rice varieties. Temperature of amplification for each primer was standardized by gradient PCR with selected rice samples. PCR reaction mixture was set to a total volume of 10 μl which contained 20 ng genomic DNA, 2 mM MgCl2, 1× buffer, 0.2 nmol each primer, 0.2 mM dNTPs, 1 unit of Taq DNA polymerase (Fermentas, Life Sciences, United States). The conditions for PCR amplification were as follows: initial denaturation at 94°C for 4 min followed by 36 cycles of 94°C for 30 s, Ta (59°C) for 45 s, 72°C for 1 min and final extension at 72°C for 10 min. PCR amplicons were separated and visualized in an automated QIAxcel gel electrophoresis system (QIAGEN) using the reagents supplied with the QIAxcel DNA High Resolution Kit. OM800 QIAxcel method was used at 5 KV sample injection voltage, 10 s sample injection time, 3 KV separation voltage and 800 s separation time for better resolution of the amplicons. Standard markers (15–500 bp QX Alignment Maker and 25–450 bp QX DNA Size Marker) were used to determine the size of amplicons with the help of QIAxcel BioCalculator (Version 3.0.05).

## Results and Discussion

### *PolyMorphPredict* Strategy for Microsatellite Mining and Primer Generation

*PolyMorphPredict* is designed for microsatellite loci mining along with its primer generation to detect polymorphic locus. This tool has six tabs, namely, Home, Primer Design, Polymorphism, Algorithm, Tutorial, and Contact. The Home page consists of a brief description of microsatellite marker, polymorphism and overview of the web server. Primer designing is the first major module of this web server, where user has to upload the genome file/chromosome wise data/transcriptome data (in fasta format) from which markers will be mined (using MISA) and primers will be designed (using *Primer3*) for those markers. MISA output file serves as the input for *Primer3* tool. *Primer3* generates five set of forward and reverse primers for each of the mined locus. For each set, it computes the respective product size and melting temperature (Tm). After completion of job, user may download the results from hyperlinks provided or provide email id for fetching the results.

### Evaluation of Polymorphism Discovery Using Various Genome Sizes

*PolyMorphPredict* was found successful in discovering the polymorphic loci with three different genome sizes. Evaluation result of larger, moderate and smaller genomes are available in Supplementary Files S1–S4. Since for polymorphism discovery in these cases, we selected simple di-nucleotide repeats, thus we got substantial number of polymorphism. Evaluation was done using simple repeat which exhibits higher degree of polymorphism than compound repeat. This is due to higher slippage rate during DNA replication (Ngai and Saitou, 2016). These analyses demonstrate the effectiveness of this tool across different genome size and also across animal and plant domain for chromosome-wise extensive microsatellite mining.

For this second major module for polymorphism detection, sub-module Self Design Primer, user has to upload two types of input files in .fasta format *namely*, the reference sequence file, which may be whole genome sequence file/chromosome-wise files/species specific files and the genotypic file. User can also select species specific genome sequence having chromosome-wise data as reference sequence. Currently, genome sequence of two crops, *namely*, rice (*Oryza sativa*) and sugarbeet (*Beta vulgaris*) are in-built in the web server. User may select chromosome-wise data for the crop. User is further given the flexibility to choose the chromosome number and their start and end position from where the markers would be mined to detect polymorphism. The list of polymorphic markers found, if any will be displayed and can be graphically visualized by clicking on the “Gel View.” The results can be downloaded from the email id provided.

Similarly, for the sub-module “External primers,” user can use primers which are already available in published literature and user can check whether these are capable of exploring the microsatellite marker polymorphism present in the genotypes or not. User need to provide “input” of the primer pairs along with the reference sequence and genotype sequence files. The output file of results will show the details of polymorphic loci, product sizes and gel view for each of the microsatellite locus specific primer pair separately.

### *In silico* Evaluation for Microsatellite Transferability Across Species

*PolyMorphPredict* was successfully used for evaluation of microsatellite transferability using e-PCR module in heterologous mode. Out of 200 microsatellites of focal species, sweet cherry (*Prunus avium*), 24 microsatellite loci were found present in heterologous host species peach (*Prunus persica*) (Supplementary File S5). This heterologous mode transferability depends on genetic closeness of the two species. Higher magnitude of transferability reflects genetic propinquity in process of species divergence (Dirlewanger et al., 2002). Since there are >700 species of *Prunus* (Depypere et al., 2007) and genome sequence of all are not available thus our tool can be further promising in prioritizing the microsatellite loci which are not only present across species but also having higher degree of polymorphism.

### Validation of *PolyMorphPredict* for Detection of Polymorphism: A Case Study in Rice

#### Polymorphism Discovery by e-PCR

Results obtained by e-PCR on four Indian rice varieties out of 36 most polymorphic markers of HvSSR (Highly variable SSR) series (three markers/chromosome) could detect only 12 loci successfully. The wet lab validation result successfully demonstrates polymorphism on selected loci. Out of 36 loci which were subjected to e-PCR, only 24 were with PCR product size (Supplementary Table S2). Remaining 12 loci could not be detected by e-PCR which might be due to missing of corresponding sequences in the re-sequence data of the respective varieties. Out of 24 detected loci, ten were found to be polymorphic and remaining 14 were either monomorphic or missing.

#### Polymorphism Discovery by *in vitro* PCR

**FIGURE 2:**
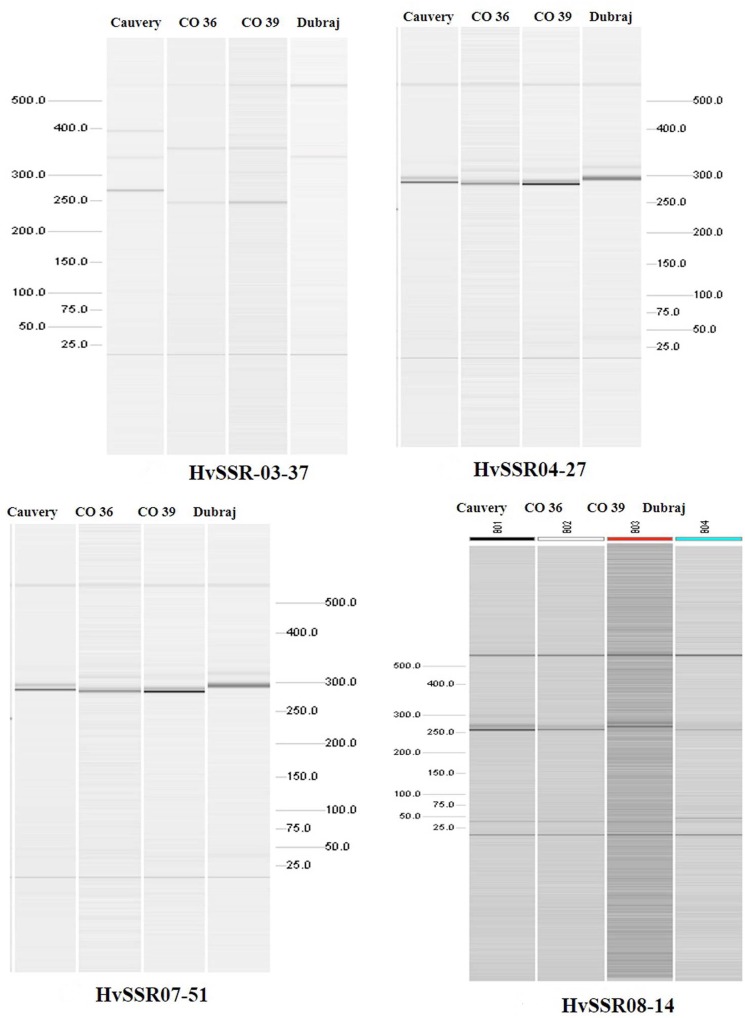
Polymorphismdiscovery by automated QIAxcel gel electrophoresis based differentiation of microsatellite alleles.

Among the ten e-PCR polymorphic loci, we selected four different microsatellite locus from different chromosomes for *in vitro* PCR validation. Allelic data generated by wet lab validation of all the four loci were in correspondence with the allelic profile obtained by e-PCR by *PolyMorphPredict*. Comparison of *in silico* (e-PCR) and *in vitro* PCR genotyping results are presented in Table 1. These wet lab result confirms the utility of this tool for rapid polymorphic discovery saving time, energy and cost (Figure 2).

**Table 1:** Comparison of rice microsatellite loci genotyping by *in silico* and *in vitro* PCR.

Microsatellite loci	Forward primer	Reverse primer	PCR genotyping	Allelic profile (bp)			
				
				Cauvery	Co36	Co39	Dubraj
HvSSR03-37	GGAAATCGTCAAGAACGTC	TAATTGTATACCACTCCGCC	*In silico*	337	351	305	338
			*In vitro* PCR	337	351	305	338
HvSSR04-27	ATGGATTTAGGCTTGTTTGA	ATACTGCGAAGGTGAAGAGA	*In silico*	292	292	293	296
			*In vitro* PCR	292	292	293	296
HvSSR07-51	CGAGCATGTCTGTCAAGTAA	GTTCGAATGTAATGTTGGCT	*In silico*	286	281	282	293
			*In vitro* PCR	286	281	282	293
HvSSR08-14	TCCACTTTACATCGTCACAA	CTACCTCTTAACCGCACATT	*In silico*	257	258	266	257
			*In vitro* PCR	257	258	266	257


This tool is designed for rapid microsatellite polymorphism discovery in genome re-sequencing projects where template reference genome is available with chromosome-wise genomic data or transcriptomic data in fasta format. As this is a bulk microsatellite mining tool intending to discover polymorphic loci thus default parameters of MISA has been used to obtain loci having higher probability of putative polymorphic region across entire chromosome. Similarly, default parameters of Primer3 has been used to have uniform Tm of all microsatellite loci for bulk genotyping. If polymorphism discovery has been done using transcriptomic data, then designed primer may be evaluated using “*External Primer.*” Such additional evaluation of primer with template reference genome user can avoid *in vitro* PCR failure, which may happen due to exon-intron junction in genomic DNA. Since the tool is giving four sets of primers for every locus, thus the chance of mutated 3′ end primer binding site leading to PCR failure can easily be resolved by use of remaining set of primers. Even chance of mutated 3′ end primer binding site leading to PCR failure can easily be resolved by use of remaining set of primers as the tool gives four sets of primers for every locus.

Often stringency parameter maneuverability is required in primer designing allowing leniency of primer mismatch and customized PCR product size on shorter DNA fragment. Such situations are frequently encountered in use of primers in heterologous species mode where null alleles are expected. Since all input sequence in bulk microsatellite polymorphism discovery are from re-sequencing of a single species, thus such situations are not expected. Moreover in eukaryotic genome these microsatellites are most abundantly distributed, thus obviating the need of such precautions. Polymorphism discovery in some plant species may face issue of paralogy where a single set of primer is having binding site over multiple chromosome. Such multilocus, multi-chromosomal genotyping will generate erroneous allelic data by exhibiting multiple bands in gel based genotyping or multiple allele number in automatic genotyper. Paralogy is often encountered in species having polyploidy or allopolyploidy (Korbecka et al., 2010). This tool can be used to resolve the issue of paralogy using “external primer” evaluation with individual chromosome/set of sub-genomes. By such evaluation of paralogus loci can be discarded before wet lab validation saving time and cost of genotyping.

Interestingly, this single server can be used to analyze both, DNA and RNA sequence data for microsatellite polymorphism discovery. Even transcriptome based genic region microsatellite can be discovered and primers can be designed for genotyping. Such genic region primers can be electronically validated by conducting e-PCR in our tool using reference genome. Thus, our tool can also be used to evaluate genotyping efficacy of genic microsatellite or putative functional domain marker primer designing. Such genic region primer must be designed avoiding intronic junctions in PCR template to generate accurate allelic data on genomic DNA of various genotypes. Such initial evaluation can save oligo-synthesis cost and avoid failure/erroneous result in wet lab validation of genic region microsatellite primers over genomic DNA. Genome-wide microsatellite makers developed and displayed graphically in our server with the options to synthesize the primers directly for the desired regions of the chromosome will serve the ease of developing markers for rapid genotyping. *PolyMorphPredict* will be useful to reduce the cost, time along with prioritization of microsatellite loci required for multiplexing in genotyping. Such rapid polymorphic locus identification along with ready to use primers for genotyping can be of immense use in ecological diversity, population genetics and evolution. For example, in case of diversity studies, they have been used in functional diversity (Acuña et al., 2012), ecological trait diversity assessment (van Tienderen et al., 2002). Molecular markers have been used in computation of important attributes of an ecological population (Putman and Carbone, 2014) like estimation of inbreeding (Al-Atiyat, 2016) and relatedness (Taylor, 2015), population viability and extinction rate estimation (Sattler et al., 2017), genealogical registrations and conservation decisions (Sereno et al., 2008). Designing conservation strategies based on Franklin’s 50/500 rule of conservation (Franklin, 1980), such tool can be used to estimate effective population size and optimal census size to rationalize and optimize time and cost factor in a conservation program formulation. The permissible limit of inbreeding (1% per generation) in such model can be monitored using microsatellite data (Lehmkuhl, 1984). microsatellite data can be used in strategy formulation required in management of endangered species (Jamieson et al., 2006) population re-introduction (Jamieson, 2015) and development of ecological model system (Wheat, 2010).

Though this tool has been developed primarily for polymorphism discovery in model species only. In case it is used for non-model species, it will have limitations like non-availability of chromosome-wise data for computational ease. Such approach of non-model use are known to have certain limitations like compromised diversity calculus, non-linear relation in divergence time estimation using genetic distance (Meglécz et al., 2012) and selection of microsatellites with biasness due to selective conserved orthologous region compromising homogenous representation of loci for genome in population variability (Li and Kimmel, 2013).

Apart from ecological and conservation applications *PolyMorphPredict* can be used in germplasm evaluation, characterization, identification, improvement and management. For example, use of polymorphic microsatellite in DUS test for variety identification and management (Yim et al., 2009), diversity analysis (Singh et al., 2013), marker assisted selection (MAS), gene assisted selection (GAS), quantitative trait locus (QTL) and gene mapping (Wu et al., 2015), germplasm improvement (Leonova et al., 2011), traceability of product/produce (Fujita et al., 2009), tracing hybridization and introgression events (Twyford and Ennos, 2012), differentiation of essentially derived variety (EDV)/initial variety (IV) in case of varietal disputes (Kwon et al., 2005), seed purity, and hybrid testing (Wang et al., 2014). *PolyMorphPredict* may also find its application in identification and differentiation of fungi, parentage testing in animals and fish, breed signature of animal and fish, genome finishing, etc.

## Conclusion

A universally applicable tool, *PolyMorphPredict* has been developed which mines polymorphic microsatellite loci and computes primers using both whole genome DNA data as well as RNA data of transcriptome of any species. It has additional provision to perform e-PCR using published or external primers for polymorphism discovery with in species and transferability across heterologous species. It can reduce the time and cost required for microsatellite polymorphism discovery without compromising accuracy and efficiency. Efficacy of this tool has been evaluated for microsatellite mining, polymorphism discovery and transferability studies using various five species having 21 genotypes. It clearly demonstrates its immense use not only in research acceleration in area of ecological diversity and evolution, population genetics analysis but can also be of much pragmatic use in germplasm management, improvement and conservation.

## Package Availability and Requirements

The *PolyMorphPredict* is freely accessible to research community for non-commercial use and available at http://webtom.cabgrid.res.in/polypred/. It operates on windows operating system. The languages used are PERL, R along with MISA and Primer3.

## Standard of Reporting

The data used in the study are from public domain, which has been mentioned along with the source in the manuscript.

## Author Contributions

DK, SJ, and MI conceived the theme of the study. VA, SJ, MI, UA, SF, and RD did the computational analysis and server development. RS did the wet lab validation. UA and SS developed the code for simulated gel. RD, VA, SJ, MI, and DK drafted the manuscript. DK, MI, SJ, and AR edited the manuscript. All authors read and approved the final manuscript.

## Conflict of Interest Statement

The authors declare that the research was conducted in the absence of any commercial or financial relationships that could be construed as a potential conflict of interest.

## Footnotes

1 http://cherry.kazusa.or.jp/#

## Supplementary Material

Table S1 List of genotypes and their identification codes used for microsatellite polymorphism discovery.

Table S2 List of 36 HvSSR loci subjected to e-PCR.

FILE S1 Evaluation of polymorphism discovery by *PolyMorphPredict* with larger genome size using species cattle.

FILE S2 Evaluation of polymorphism discovery by *PolyMorphPredict* with moderate genome size using species sugarbeet.

FILE S3 Evaluation of polymorphism discovery by *PolyMorphPredict* with smaller genome size using species rice.

FILE S4 Evaluation of polymorphism discovery by *PolyMorphPredict* with smaller genome size using species grape.

FILE S5 Evaluation of between species microsatellite transferability by *PolyMorphPredict* using genus *Prunus.*

## Abbreviations

DNA: deoxyribonucleic acid
DUS: distinctiveness, uniformity and stability
EDV: essentially derived variety
GAS: gene assisted selection
IP: intellectual property
IV: initial variety
MAS: marker assisted selection
MISA: microsatellite analysis tool
PCR: polymerase chain reaction
QTL: quantitative trait locus
RNA: ribonucleic acid
SSR: simple sequence repeats
