# Supplementary material for: PolyMorphPredict: A Universal Web-Tool for Rapid Polymorphic Microsatellite Marker Discovery From Whole Genome and Transcriptome Data
Source: Front Plant Sci. 2019 Jan 11;9:1966. doi: 10.3389/fpls.2018.01966 (PMC6337687; doi:10.3389/fpls.2018.01966)
Supplement: FILE S4 — Evaluation of polymorphism discovery by PolyMorphPredict with smaller genome size using species grape. [file Table_6.DOCX]

***PolyMorphPredict:* A universal web-tool for rapid polymorphic microsatellite marker discovery for whole genome and transcriptome data**

Ritwika Das^1,#^, Vasu Arora^1,#^, Sarika Jaiswal^1^, MA Iquebal^1^, UB Angadi^1^, Samar Fatma^1^, Rakesh Singh^2^, Sandip Shil^3^, Anil Rai^1^, Dinesh Kumar^1,*^

^1.^ Centre for Agricultural Bioinformatics, ICAR-IASRI, New Delhi-110012, India

^2.^ ICAR-National Bureau Plant Genetic Resources, New Delhi-110012, India

^3.^ Research Center, ICAR-Central Plantation Crops Research Institute, Mohitnagar, Jalpaiguri, West Bengal, India -735102

*Corresponding Author: [dinesh.kumar@icar.gov.in](mailto:dinesh.kumar@icar.gov.in)

^#^ Contributed Equally

**Supplementary Table 1.** List of genotypes and their identification codes used for microsatellite polymorphism discovery

| **Genotypes/ Cultivars** | **Assembly / Accession / BioProject / SRA number** |
| --- | --- |
| **Grape *(Vitis vinifera)*** | |
| PN40024 | GCA_000003745.2 |
| Chkhaveri | GCA_002922885.1 |
| Saperavi | GCA_002923015.1 |
| Meskhetian green | GCA_002923105.1 |
| Rkatsiteli | GCA_002923165.1 |
| **Prunus species** | |
| Peach (*Prunus persica*) | GCF_000346465.2 |
| Sweet cherry (*Prunus avium*) | GCA_002207925.1 |
| **Sugarbeet (*Beta vulgaris* ssp. *Vulgaris)*** | |
| KWS2320 | RefBeet-1.1, RefBeet-1.2, RefBv |
| KWS230 DH1440 | [KDHBv](http://bvseq.boku.ac.at/Genome/Download/KDHBv/) |
| STR06A6001 | [UMSBv](http://bvseq.boku.ac.at/Genome/Download/UMSBv/) |
| SynMono | [YMoBv](http://bvseq.boku.ac.at/Genome/Download/YMoBv/) |
| SynTilling | [YTiBv](http://bvseq.boku.ac.at/Genome/Download/YTiBv/) |
| **Rice (*Oryza sativa*)** | |
| Dubraj | ERX591655 |
| Co-39 | SRX1502127 |
| Co-36 | ERX562686 |
| Cauvery | ERX571499 |
| RP Bio-226 | GCA_001305255.1 |
| Shuhui498 | GCA_002151415.1 |
| **Cattle (Bos taurus)** | |
| Hereford | NC_037328 |
| Nelore | 9915_ref_Bos_indicus_1.0_chr1 (1).fa (PRJNA72827) |
| Gir | PRJNA427256 |
