# Supplementary material for: PolyMorphPredict: A Universal Web-Tool for Rapid Polymorphic Microsatellite Marker Discovery From Whole Genome and Transcriptome Data
Source: Front Plant Sci. 2019 Jan 11;9:1966. doi: 10.3389/fpls.2018.01966 (PMC6337687; doi:10.3389/fpls.2018.01966)
Supplement: FILE S5 — Evaluation of between species microsatellite transferability by PolyMorphPredict using genus Prunus. [file Table_7.docx]

***PolyMorphPredict:* A universal web-tool for rapid polymorphic microsatellite marker discovery from whole genome and transcriptome data**

Ritwika Das^1,#^, Vasu Arora^1,#^, Sarika Jaiswal^1^, MA Iquebal^1^, UB Angadi^1^, Samar Fatma^1^, Rakesh Singh^2^, Sandip Shil^3^, Anil Rai^1^, Dinesh Kumar^1,*^

^1.^ Centre for Agricultural Bioinformatics, ICAR-IASRI, New Delhi-110012, India

^2.^ ICAR-National Bureau Plant Genetic Resources, New Delhi-110012, India

^3.^ Research Center, ICAR-Central Plantation Crops Research Institute, Mohitnagar, Jalpaiguri, West Bengal, India -735102

*Corresponding Author: [dinesh.kumar@icar.gov.in](mailto:dinesh.kumar@icar.gov.in)

^#^ Contributed Equally

**Supplementary Table 2.** List of 36 HvSSR loci subjected to e-PCR

| **Sl. No.** | **Locus** | **Forward** | **Reverse** | **Product (*in silico* in reference genome)** | **Cauvery** | **Co36** | **Co39** | **Dubraj** |
| --- | --- | --- | --- | --- | --- | --- | --- | --- |
| 1 | HvSSR01-32 | AAACTGGAGATGAACTCGAA | GTAACGAACTAGAGCATGGG | 250 | 0 | 250 | 0 | 0 |
| 2 | HvSSR01-41 | TGAGTGAGACTTGACAGTGC | AGTTAACACCAATGCTGACC | 348 | 0 | 0 | 0 | 348 |
| 3 | HvSSR01-53 | TGTCGTCCACGTAGTAGGAG | ACACTCCTCCTCTGTTCTCA | 274 | 0 | 0 | 274 | 274 |
| 4 | HvSSR02-01 | AAGAGATGAGAAGAGCAATGA | CAACTTAGAGGAAGAAGGAGG | 312 | 0 | 312 | 0 | 0 |
| 5 | HvSSR02-33 | TAATGCACGCACAACTTTAC | TATAGAATGCTGACTGGGCT | 355 | 0 | 0 | 0 | 0 |
| 6 | HvSSR02-50 | TTTCAGGAATCTGATGCTTT | TTAATCAAAGCCCTAACAGC | 195 | 0 | 195 | 0 | 195 |
| 7 | HvSSR03-02 | TAGCGGAGTTGGAATAACAC | CTGCACTGCATACCTCATAA | 228 | 0 | 0 | 0 | 228 |
| 8 | HvSSR03-10 | GTACACAACGTCACAACAGC | ACTGTGGCATATGTTCGATT | 280 | 0 | 0 | 0 | 0 |
| 9 | HvSSR03-19 | AATTCAGTTCACGCATTCTT | AGCTGTTCGTCTGCATAGTT | 238 | 238 | 238 | 238 | 0 |
| 10 | HvSSR03-37 | GGAAATCGTCAAGAACGTC | TAATTGTATACCACTCCGCC | 386 | 337 | 351 | 305 | 338 |
| 11 | HvSSR03-54 | GCCTATCAGGCTATCATCAC | GTGATCGACATTGAGGAGTT | 352 | 351 | 0 | 0 | 0 |
| 12 | HvSSR04-19 | TCGTGGAGTATCCTGTATCC | TTATAACTTGGAGCTCAGGC | 265 | 0 | 0 | 0 | 0 |
| 13 | HvSSR04-27 | ATGGATTTAGGCTTGTTTGA | ATACTGCGAAGGTGAAGAGA | 318 | 292 | 292 | 293 | 296 |
| 14 | HvSSR04-46 | GGCGCGCTTATATATGTACT | CGATTGCGTGGTGTAACTAT | 179 | 0 | 0 | 178 | 0 |
| 15 | HvSSR05-09 | CTCTCCATCTTGCAATCTTC | TGCATGACTCTATCAACCAG | 335 | 0 | 0 | 0 | 0 |
| 16 | HvSSR05-15 | CCATGTCAAACGGTTACTTT | GGGAGAAGTGAGAAAGAGGT | 275 | 0 | 274 | 0 | 274 |
| 17 | HvSSR05-30 | TACGACGGACGATTAAAGTT | GCTAACTCATTCATCTCGCT | 353 | 0 | 0 | 0 | 0 |
| 18 | HvSSR06-03 | CTAGGGAATCAGCGGTTAG | GCTCTCTTGTCCTTCTTCTTC | 217 | 0 | 217 | 0 | 0 |
| 19 | HvSSR06-16 | TCTGAAATGCTGTCATCAAG | GAGCAGAGTAGGACATGAGC | 368 | 368 | 368 | 368 | 368 |
| 20 | HvSSR06-40 | CTCTTCCGTGGTTAAAGAAA | CACTGGTATGATCTCCGACT | 385 | 0 | 0 | 0 | 0 |
| 21 | HvSSR07-18 | GGTGTGTTGTCGAATCTCTC | ATGCCATTGTCCTTACATTC | 343 | 0 | 0 | 0 | 0 |
| 22 | HvSSR07-51 | CGAGCATGTCTGTCAAGTAA | GTTCGAATGTAATGTTGGCT | 341 | 286 | 281 | 282 | 293 |
| 23 | HvSSR08-14 | TCCACTTTACATCGTCACAA | CTACCTCTTAACCGCACATT | 295 | 257 | 258 | 266 | 257 |
| 24 | HvSSR08-19 | CATCTCTTGAGAAATCTGCC | TGTGCATTTCGTCTTTCATA | 221 | 0 | 0 | 0 | 0 |
| 25 | HvSSR09-11 | TGCAGAATTTCTTCCTTCAT | ACCAGAATCTCCCAAATGTA | 366 | 366 | 366 | 0 | 0 |
| 26 | HvSSR09-26 | TGGGCATCTGGTACTATCTT | AGCTCATTCCACAGGTTAGA | 331 | 331 | 331 | 331 | 330 |
| 27 | HvSSR09-55 | TTACTCCGCATATATCCATGT | ATTTGACACCAAGTTGATCC | 1367 | 0 | 0 | 0 | 0 |
| 28 | HvSSR10-03 | TCTTTCCCAAATTCCAGATA | CATTAGTTGTTTGTGGCAGA | 289 | 289 | 289 | 289 | 289 |
| 29 | HvSSR10-13 | CAGGGAATCAACATCAAAGT | AGCAAGGCAAGTCATCTCTA | 169 | 0 | 0 | 0 | 0 |
| 30 | HvSSR10-34 | TAGACCGAGGAATTGAAAGA | TTTGGGCTTATTGTCAGTTT | 202 | 0 | 202 | 0 | 0 |
| 31 | HvSSR11-13 | TGAAACCACAATGAGTCAAA | GCCCTAAACCCAAATAGAAG | 285 | 0 | 285 | 0 | 0 |
| 32 | HvSSR11-21 | TACGCTATAACCATGAAGCA | CTCCCGTTATTGTCCTTACA | 291 | 0 | 291 | 291 | 0 |
| 33 | HvSSR11-58 | ACTGAATCCTTACTGGAGCA | GGAGATAAGCATTTGGAAGA | 371 | 0 | 0 | 0 | 0 |
| 34 | HvSSR12-01 | GATTTGCAACACGTACGATA | GATCATCCACTCTGAGCAAT | 271 | 0 | 0 | 0 | 0 |
| 35 | HvSSR12-13 | ACCTTAGGGCTGAGTTCTTT | TTAGGCTTGTCTCTTCCTCA | 388 | 388 | 388 | 389 | 388 |
| 36 | HvSSR12-39 | ATCTAACAACAACAATCCCG | CATCTTCATCCCTCGTGTAT | 289 | 290 | 0 | 0 | 0 |
